# Environmental Determinants of Type 1 Diabetes: From Association to Proving Causality

**DOI:** 10.3389/fimmu.2021.737964

**Published:** 2021-10-01

**Authors:** Lauren M. Quinn, F. Susan Wong, Parth Narendran

**Affiliations:** ^1^ Institute of Immunology and Immunotherapy, Research College of Medical and Dental Sciences, University of Birmingham, Birmingham, United Kingdom; ^2^ Diabetes Research Group, Division of Infection and Immunity, School of Medicine, Cardiff University, Cardiff, United Kingdom; ^3^ Department of Diabetes, University Hospitals of Birmingham NHS Foundation Trust, Birmingham, United Kingdom

**Keywords:** type 1 diabetes (T1D), seroconversion, auto-antibodies, autoimmunity, environmental factors, gut micro biome, obesity, infection - immunology

## Abstract

The rising incidence of type 1 diabetes (T1D) cannot be ascribed to genetics alone, and causative environmental triggers and drivers must also be contributing. The prospective TEDDY study has provided the greatest contributions in modern time, by addressing misconceptions and refining the search strategy for the future. This review outlines the evidence to date to support the pathways from association to causality, across all stages of T1D (seroconversion to beta cell failure). We focus on infections and vaccinations; infant growth and childhood obesity; the gut microbiome and the lifestyle factors which cultivate it. Of these, the environmental determinants which have the most supporting evidence are enterovirus infection, rapid weight gain in early life, and the microbiome. We provide an infographic illustrating the key environmental determinants in T1D and their likelihood of effect. The next steps are to investigate these environmental triggers, ideally though gold-standard randomised controlled trials and further prospective studies, to help explore public health prevention strategies.

## Introduction

An estimated 1.1 million people under 20 years of age are affected by type 1 diabetes (T1D) worldwide (1, 2). T1D represents 5-10% of the global diabetes burden (3) and is not a disease of childhood alone, with almost half diagnosed in adulthood (4–6). Overall annual increase in T1D is estimated at 3% (2-5%) (1, 7), with rising trends observed across all age groups over the last three decades (1, 3). Some of the greatest increases are observed in historically low prevalence countries (4, 8).

T1D is a chronic autoimmune condition, characterised by hyperglycaemia and long-term insulin dependency (9). T1D pathophysiology is defined by three stages of disease progression (10). Stage one is seroconversion to one or more autoantibodies (10), including glutamic acid decarboxylase (GAD), anti-insulin, insulinoma-associated antigen 2 (IA2) and zinc-transporter 8 (Zn-T8). Presence of two or more antibodies will see 70% of children develop T1D in the next 10 years, whilst four autoantibodies invariably confer 100% risk (11). Stage 2 is damage to the beta-cells causing pre-symptomatic dysglycaemia and stage 3 is overt T1D due to beta-cell failure with requirement for exogenous insulin (10). This provides different targets for prevention at the stages of primary prevention (preventing seroconversion, in those genetically at risk), and secondary prevention (preventing loss of and damage to the beta-cells in individuals with autoimmunity/autoantibodies) (12).

The primary risk factor for T1D is genetic. It is strongly associated with HLA-DR3-DQ2 and/or HLA-DR4-DQ8 haplotypes (13). The significance of genetics is further evidenced by the increased risk observed where a sibling (8%), father (5%) or mother (3%) has T1D. The major histocompatibility complex (MHC) encoding the HLA region confers 50% of the genetic risk for T1D (13). Genome-wide association studies have identified an additional 50 loci that confer susceptibility (13).

However, genetics alone does not equate to causality and an array of environmental factors are implicated to trigger T1D seroconversion and disease progression (14). The threshold hypothesis describes a model in which genetic and environmental factors represent intersecting and reciprocal trend lines, defined by odds ratios in rank order, which cumulatively confer risk of progression to T1D until a critical threshold is met (15).

Evidence to support the nurture side of the argument for T1D continues to expand. Firstly, the rising incidence of T1D in recent decades is too rapid to be explained by genetic changes alone (8). Secondly, T1D concordance rates between monozygotic twins is <50% (16). Thirdly, T1D incidence also demonstrates significant geographic and latitudinal differences, with higher incidence in Nordic countries (17–20), and migration studies show that the incident risk of the new location is assumed (21). Fourthly, incidence is increasing across all age groups, and the incidence in younger children is rising (1), despite highest risk genotypes declining over the last 20-40 years (22, 23). The highest rates are observed in previously low incidence countries and countries with the highest economic growth (8).

It may be more than coincidence that T1D is therefore a heterogenous condition determined by a combination of genetic, immunological, and metabolic factors, and this complexity reflects the array of environmental triggers implicated in the pathogenesis.

Numerous other reviews have explored environmental contributions to T1D (8, 12, 14, 24, 25). These have largely outlined association studies between different environmental determinants and the development of T1D. Some of these reviews also predate the results of the seminal, ‘The Environmental Determinants of Diabetes in the Young’ (TEDDY) study, from which different aspects have been published over the last 6 years (26).

In this narrative review, we explore the putative environmental risk factors for T1D with an emphasis on testing causality. Since causality is best tested in the setting of a double-blind randomised controlled trial (RCT), we outline the different RCTs in this area. As part of the review, we outline potential underlying mechanisms to the different environmental determinants and the stage of pre-T1D at which they could exert an influence.

We undertook this review though searching PubMed and Medline. We used the following search terms ‘environment AND type 1 diabetes’; type 1 diabetes (T1D) OR islet autoimmunity (IA) combined with the following - enterovirus; rotavirus; influenza; COVID-19; vaccine OR vaccination; birth weight; weight gain; BMI; childhood obesity; gut microbiome OR gut microbiota; diet; breast milk OR breastfeeding; cow’s milk; formula milk; gluten; antibiotic; probiotic; vitamin D; and nicotinamide; omega-3. We included systematic reviews, meta-analyses, RCT, cohort studies (prospective and retrospective) and case-control studies. We have included the largest retrospective and prospective European cohort and case control studies, including ‘Type 1 Diabetes Prediction and Prevention’ (DIPP) (27), ‘Early Childhood Diabetes in Finland’ (DiMe) (28), ‘Danish National Birth Cohort’ (DNBC), ‘Norwegian Mother and Child cohort’ (MOBA) (29), ‘Diabetes Autoimmunity Study in the Young’ (DAISY) (30), DIABIMMUNE, Environmental Triggers of T1D (MIDIA - Norwegian acronym) (31), Finnish Dietary Intervention Trial for the Prevention of T1D (FINDIA) (32), TEDDY study (26), and trials including ‘Trial to Reduce IDDM in the Genetically at Risk’ (TRIGR) (33), the ‘European Nicotinamide Diabetes Intervention Trial’ (ENDIT) (34), and the Deutsche Nicotinamide Intervention Study (DENIS) (35). Reference lists were also screened for relevant articles.

## Infections and Vaccinations

Viruses are important contenders for environmental triggers of T1D (36, 37).

Acute fulminant diabetes, termed type 1b diabetes, is reported following infection with Mumps, Coxsackie B3 and B4, Rubella, and Influenza B infection (38). The hyperglycaemic ketosis is sudden, symptoms occur for one week, islet antibodies are negative, and C-peptide is extremely low. In this instance, beta-cell damage occurs secondary to direct lytic effects from viral invasion, causing widespread beta-cell destruction and absolute insulin deficiency, without autoimmunity (37, 38).

An alternative association, expanded below, appears to be a more chronic, repeated viral exposure. Implicated mechanisms here include molecular mimicry, where viral epitope sequences bear resemblance to beta-cell antigens and potentially trigger a cross-reactive autoimmune response (36). Viral infection of beta-cells will also result in over-expression of MHC class I, resulting in presentation of self-antigens and perpetuation of further autoimmunity (36, 37, 39). Viruses implicated in this more chronic-repeated infection model are outlined below.

### Enterovirus

The most robust evidence for a viral trigger exists for enteroviruses (EV) (40–42). T1D incidence correlates with enteroviral infection rates and the seasonal variation in T1D is preceded by enteroviral epidemics (41, 43). However, EV is a common childhood infection, hence HLA susceptibility to T1D, combined with genetically determined susceptibility and inflammatory response to EV are critical determinants of risk (41, 44–46). EV infection potentially initiates and accelerates all three stages of T1D pathophysiology (42, 43).

EV spreads and replicates *via* the upper respiratory and gastrointestinal tracts, and invades the beta islet cells *via* the coronavirus adeno receptor (CAR) (36). Inefficient viral clearage of EV (47) and induction of a chemokine response from the beta-cells triggers islet autoimmunity (IA) through molecular mimicry, inflammation, bystander effects, and T cell suppression (48). EV chronicity appears critical to sustain beta-cell autoimmunity; repeated infection with EV strains further increases risk (36).

Systematic reviews (41, 49) and cohort studies (27, 28, 50, 51) demonstrate positive associations between persistent EV infection, autoimmunity, and progression to stage 3. The DiMe study (28) and the DIPP (27) study showed that EV infection in pregnancy or early childhood respectively increased risk of T1D. Interestingly, studies show evidence of seroconversion to islet autoantibodies in both mother postnatally and child, following enteroviral infection during pregnancy (52, 53). A meta-analysis showed that maternal infections were also significantly associated with T1D progression, and most notably for maternal enterovirus infections, odds ratio (OR) 1.54 (confidence interval (CI) 1.05-2.27) (54). The postulated mechanism is transfer of epitopes/molecular mimicry that triggers autoimmunity in the offspring, but the supporting evidence is limited. Cross-reactivity between EV with GAD and IA2 epitopes could trigger seroconversion, but equally, secondary to chronic and cumulative viral infections, primed auto-reactive T cells could promote progression to stage 2, in auto-antibody positive individuals (36).

Most recently, the TEDDY study showed from a genetically predisposed cohort of children (n=8676) followed-up over 15 years, that chronicity of Coxsachie B (EVB), from persistent shedding in the stool, predicted development of IA, particularly anti-insulin antibodies (51, 55, 56). Conversely, acute EVB infection, without prolonged stool shedding, did not associate with autoimmunity or T1D in this cohort (51). A systematic review and meta-analysis by Yeung et al. (41) further showed that persistent EVB increases risk of IA and T1D with an OR of 3.7 (CI: 2.1-6.8) and 9.8 (5.5-17.4) respectively. Problems remain in detecting the specific strain(s) of EV that confer the highest risk.

Based on this strong association between chronic EV infection and T1D (51, 56), a RCT with a polyvalent vaccine to EV is currently planned. An EV vaccine would target primary and secondary prevention by reducing recurrent EV infection, limiting exposure to potential reactive epitopes and chronic beta-cell inflammation that could contribute to IA (48, 57).

### Rotavirus

Introduction of the childhood rotavirus (RV) vaccination in Australia, and the fall in T1D incidence that followed, was postulated to implicate a causal environmental trigger (58). The outer capsid protein of the human RV (VP7) shares 56% identity and 100% similarity with a dominant epitope in IA2, and both bind to (HLA-DR4) (*0401) (59), suggesting molecular mimicry and cross-reactive T cells as mediators of IA (59, 60). However, the DIPP longitudinal cohort study exploring RV in T1D pathophysiology failed to show an association between RV and auto-antibodies (61). A study demonstrated that RV infection prior to 6 months of age was significantly associated with human and bovine-insulin binding antibodies, but this was strongest in children receiving cow’s milk prior to 3 months (62), representing an important confounding factor.

An Australian interrupted time series analysis found a 15% T1D risk reduction in children aged 0-4 years receiving the RV vaccine compared to those not vaccinated (58). However, the Finnish population-based study with 11–14 year follow-up did not replicate these findings (63, 64). Although Rogers et al. (65) found a 33% reduced risk of T1D in vaccinated compared to unvaccinated children, Burke et al. found no association between RV vaccination and T1D incidence in a US cohort of children with commercial insurance (66). Similarly, Glanz et al. (67) found no association. Hence the evidence supporting RV vaccination as a protective environmental factor is inconclusive.

### Influenza

Studies investigating the impact of the influenza infection on stage 1 and 2 T1D risk, have also been inconclusive. Valdes et al. (68) and Nenna et al. (69) showed increased risk of T1D following influenza infection whereas Kondrashova et al. (70) showed no increased risk in children genetically susceptible to T1D.

With regard to vaccination, Ruiz et al. (71) and Bardage et al. (72) showed no association between T1D risk and influenza vaccination. The Pandemrix vaccine, which caused narcolepsy in genetically susceptible individuals, raised concerns for T1D cross-reactivity and was investigated in the TEDDY study in a Finnish and Swedish cohort (73). Here, the Pandemrix vaccine did not increase risk of seroconversion for one or two islet autoantibodies [HR 0.75 (0.55-1.03) and HR 0.85 (0.57-1.26)] respectively, after adjusting for confounders (73). Interestingly, 73% received a second dose in Sweden compared to 0.6% in Finland, and the Finnish cohort had a lower risk of IA; one antibody [HR (0.47 (0.29-0.75)], or more than one antibody [HR 0.50 (0.28-0.90)], and risk of T1D [HR 0.38 (0.20-0.72)] (73). The TEDDY study is ongoing to explore links between influenza vaccination and progression to stage 3 T1D, but no RCTs are planned to test the associates listed above.

### COVID-19

Individuals with T1D are at greater risk of the Sars-CoV2 coronavirus (COVID-19) and more susceptible to severe infection (74). Global studies have suggested an increased incidence of T1D during the COVID-19 pandemic and higher frequency of presentation in severe diabetic ketoacidosis (DKA) (37, 75–78). However, important confounders include delay in seeking medical assistance and the resulting later presentations in DKA (37, 75, 78), as well as the high numbers of patients with type 2 diabetes presenting in DKA. The latter is evidenced by cases of COVID-19 associated DKA who were eventually weaned off insulin onto oral therapies (79, 80).

The coronavirus gains access to lung and gut epithelium *via* the angiotensin converting enzyme-2 (ACE2) functional receptor, which is also highly expressed in islet cells (81–83). The previous SARS-CoV1 2003 epidemic was associated with elevated fasting plasma glucose, with hyperglycaemia as an independent predictor of morbidity and mortality, even in mild pneumonitis cases with no steroid requirement (84–86). Similarly, in SARS-CoV2, hyperglycaemia in non-diabetic patients is attributed to the inflammatory response and cytokine activation, in addition to viral infection of beta-cells, which decreases insulin production (37, 86). It remains unclear if COVID-19 is simply triggering a fulminant diabetogenic state or presents the final trigger for T1D progression. However, in the former scenario, the beta-cell damage has not always persisted, evidenced by cases that were weaned off insulin and onto oral therapies (79, 80). Moving forward, the CoviDiab study (87) and roll-out of COVID-19 vaccination programmes (88) may help delineate causal relationships between COVID-19 and T1D risk.

### Vaccinations

The steady rise in autoimmune and allergic diseases in industrialised countries has been linked to a reduction in infectious diseases. Contributing factors include geography and climate (North-South gradient), childhood mixing and subsequent exposure to childhood infection (20), and vaccination programmes, and this relationship underlines the hygiene hypothesis. The incidence of T1D is lower in countries without nationwide vaccination programmes and contrasts starkly to countries with established (or newly implemented) vaccination programmes, where there is a rising T1D incidence (8). *In vivo* studies have shown in nonobese diabetic mice for T1D models, T1D incidence is higher in the mice bred in specific pathogen free (SPF) conditions compared to those bred in conventional facilities (20, 89, 90).

In terms of vaccinations being directly linked to T1D, cohort studies and meta-analysis performed to explore a causative association have failed to identify a link to date (91, 92). Childhood vaccinations for measles, rubella, mumps, pertussis, Bacillus Calmette-Guerin (BCG), Haemophilus influenza B (HiB), Tetanus Diptheria poliomyelitis, Measles/Mumps/Rubella and Diptheria/Tetanus/Pertussis showed no association with T1D, harmful or protective (91). Kühtreiber et al. performed a randomised 8 year study in T1D and showed that three years after receiving two doses of the BCG vaccine, HbA1c was lowered to near normal levels for five years (93). The mechanism was demonstrated *in vitro* and *in vivo*, and can be explained by a metabolism shift from oxidative phosphorylation to aerobic glycolysis, the latter of which is a high glucose usage state. The BCG vaccine also had a role in re-programming tolerance, through epigenetic demethylation of regulatory T cell signature genes, resulting in upregulation of mRNA expression and subsequent induction of T regulatory cells (93).

### Infections and Vaccinations – A Summary

The evidence of viral infections as a risk factor for T1D is strong. The strongest evidence appears to be through a direct lytic effect on beta-cells (37), for example following infection with mumps. Alternatively, the more insidious classical autoimmune T1D appears to have the strongest association with EV infection (36) and RCT involving vaccination programmes to test a causative link are in development. Associations between T1D and RV or COVID-19 need further evidence - either way there are other major population benefits to vaccinating against these two diseases. There is currently no evidence that any of the childhood vaccination programmes associate with T1D risk.

## Birth Weight, Infant Growth, and Childhood Obesity

Worldwide prevalence of obesity has risen to 5.6% in girls and 7.8% in boys (94), which is a 10-fold rise in four decades (8, 95–98). The roots lie in genetic, epigenetic, and environmental factors (99, 100). We have observed a rising incidence of childhood obesity along with that of T1D, depicting a double diabetes state which combines features of autoimmunity with insulin resistance (8, 99, 101). The obesity induced insulin resistance in children, increases the burden on the islets cells and potentially initiates, and accelerates the autoimmune processes in genetically predisposed individuals (102, 103). This has been termed the accelerator hypothesis (102, 103). Adiposity also contributes to systemic chronic inflammation, which together contributes to beta-cell damage and apoptosis, and progression to stage 2 and 3 of T1D (104).

It has been suggested that the stress on these beta-cells, either through inflammation or metabolic demand risks derailing stringently controlled events relating to protein transcription, translation or folding (26, 105–107). The aberrant proteins and peptides resulting from this “beta-cell stress”, in conjunction with local inflammation, are then capable of stimulating an autoimmune response (108–110).

### Birth Weight

Higher birth weight and infant growth rate contribute to T1D pathogenesis. A systematic review and meta-analysis by Harder et al., comprising 2.4 million children, found high birth weight (>4kg) increased risk of T1D by 17% (1.09-1.26) (111). The Danish National Birth Cohort (DNBC) and Norwegian Mother and Child cohort (MoBa) comprised 99,832 children and found an increased birth weight up to 12 months of age was significantly associated with T1D risk [HR 1.24 (1.09-1.41)] (29). Similarly, the Goldacre UK population study found that children with higher birth weight (3.5-<4kg and 4-5.49kg) compared to medium birth weight (3-3.49kg) had higher incidence of T1D, HR 1.13 (1.03-1.23) and HR 1.16 (1.02-1.31) respectively (112).

### Infant Growth and Body Mass Index (BMI)

The TEDDY study showed that higher infantile weight gain was associated with increased risk of IA [HR 1.09 per 1 kg/year (1.02-1.17)] (113). In children with first autoantibody GAD, there was also an increased risk of progression from IA to overt T1D [HR 2.57 per 1 kg/year (1.34-4.91)] (113). Increased progression from IA to T1D was also observed when height-growth pattern was lower in infancy, but higher in early childhood (113). Interestingly, Yassouridis et al. (114) used pooled analyses to show that IA was only linked with rising BMI up to three years of age, in non-diabetic mothers (adjusted OR 2.02 (1.03-3.73) (114). Finally, in the TRIGR study, although annual growth did not associate with autoantibody status, being overweight at 2-10 years of age increased risk (HR 2.39 (1.46-3.92) of progression to T1D (stage 2-3) but not risk of seroconversion (stage 1) (33).

A meta-analysis showed a positive dose response relationship between childhood BMI and T1D risk, OR 1.25 (1.04–1.51) (115). A Mendelian randomisation study found an OR of 2.76 (1.40-5.44) for T1D risk (116). Further, a Danish study showed that higher BMIz score at 7 years [OR 1.23 (1.09-1.37)] and 13 years [OR 1.20 (1.04-1.40)] of age was associated with an increased risk of T1D (117). The TrialNet pathway to prevention study found no link between BMI, BMI percentile, insulin resistance of progression to T1D (118), although Ferrara et al. showed that cumulative excess BMI was associated (119).

Given birth weight (111), body weight (115) and weight gain (113) correlate with T1D risk at an early stage, and this excess weight is most amenable to intervention in early childhood (120), this justifies early recognition and treatment. However, to date there are limited studies exploring whether reducing obesity decreases T1D risk. A number of studies have explored whether exercise programmes that reduce the insulin resistance and weight associated with obesity also reduce T1D. Exercise in both the NOD mouse model of T1D (121–124) and in people newly diagnosed with T1D appear to preserve beta-cell function (125), with evidence of reduced immune cell inflammation and insulitis in the former model (126, 127).

### Birth Weight, Infant Growth, and Obesity – A Summary

There is now reasonable evidence that increased birth weight (111), early weight gain (113) and obesity in children (115) matters, and associates with risk of IA as well as overt T1D, i.e. that this environmental factor may act to progress people into stage 1, 2, and stage 3 pre-T1D (51, 112, 113, 115). Unfortunately, RCT evidence to test causality are lacking. Surrogate studies demonstrating that exercise interventions can preserve beta-cells at stage 3 T1D do however show promise (125).

## The Gut - Microbiome and Diet

### The Gut Microbiome

The gut microbiota, established in early life, is influenced by perinatal factors and nutrition, and modulates the innate and adaptive immune systems (128, 129). Signature profiles of gut flora are observed in those genetically predisposed to, and incident cases of, T1D (130). The hallmark characteristics are decreased bacterial diversity, reduced microbiota stability, increased frequency of *Bacterioides* species and decreased frequency of P*revotella*, *Bifidobacteria*, and *Lactobacillus*, the latter of which confer immunomodulatory properties through production of short chain fatty acids (SCFA) (130). The gut microbiome potentially contributes to beta-cell autoimmunity through enhanced intestinal inflammation, increased permeability, loss of barrier function, and subsequent exposure to dietary antigens (129).

The TEDDY study used 16S ribosomal ribonucleic acid (rRNA) and metagenomic sequencing to reveal gut taxonomy from stool samples of healthy controls compared to genetically at-risk children, aged 3-46 months (131). Weak associations were identified between the gut microbial taxonomies and progression from stages 1-3 of T1D (seroconversion or progression to overt T1D) (131). Conversely, Vatanen et al. (132) showed the gut microbiome in healthy controls expressed genes which stimulated fermentation and synthesis of SCFA, although taxonomy did not significantly differ from the case subjects. This reflects functionally protective properties among healthy gut flora which are lost in predisposed and seroconverted individuals (132). The DIABIMMUNE study, which included 1000 genetically predisposed newborns, showed reduction in gut microbial diversity when progressing from autoantibody positivity to T1D. In autoantibody positive subjects, gene expression was shifted to enhance sugar transport and reduce amino acid biosynthesis, compared to non-seroconverters (31). The ABIS study, included 17,000 babies from Sweden born between 1997 and 1999 followed-up for 12 years, and showed that HLA haplotype determines gut microbial composition (133). Zhao et al. compared serial faecal samples in 11 seroconverted cases (5 of whom developed T1D) compared to controls, and found a higher bacteriophage Shannon diversity index in the controls, and these differences increased with age (134). Random Forests analysis revealed T1D-associated viral bacteriophage contigs, separate from the age-associated bacteriophage contigs, which were linked to gut microbial composition. The best predictive contig had nucletoid sequence homolog consistent with B.dorei (134). Overall, the gut microbiome is a key window to IA.

In the TEDDY study, mode of birth delivery was an important determinant of gut taxonomy in the first year of life (131). Mode of delivery cultivates the neonatal gut microbiome, which determines microbial composition, succession and function, and contributes to risk of autoimmune disease, allergy and obesity in later life (130). Vaginal delivery leads to neonatal gut colonisation that reflects the vaginal flora, comprising *Lactobacillus* and *Bifidobacterium* (130). The TEDDY study suggests that vaginal delivery leads to *Bacteroide* colonisation, which supports gut maturation and enhances microbial diversity (130, 131). Alternatively, delivery by caesarean section (CS) is associated with gut microbiota seeded from the mothers skin commensals, namely *Clostridium* and *Staphylococcal* species (130). The lack of colonisation by *Lactobacillus* and *Bifidobacterium species* confers dysfunctional immunomodulation with resultant implications for autoimmunity (130, 131). Risk of T1D following CS vs vaginal delivery was evaluated in a meta-analysis, including 20 studies. After adjustment for confounders, CS increased risk of T1D by 23% (1.15-1.32) compared to vaginal delivery (135). Another systematic review, comprising 9 observational studies and including 5 million births found that elective CS increased childhood T1D risk by 12% (1.05-1.20) compared to vaginal delivery. However, following adjustment, risk differences did not remain due to large study heterogeneity (136). Separate analyses focussed on cohort studies, which reduced the heterogeneity and showed T1D risk was significantly higher in elective CS [OR 1.12 (1.01-1.24)]. In contrast, the DIPP (137) and DIABIMMUNE (132) studies both demonstrated higher levels of *Bacteroide* colonisation in genetically predisposed children who seroconverted and progressed to T1D, contrary to evidence that early colonisation with *Bacteroidetes* species comprised healthy gut flora (130). The gut microbiome is therefore complex, and we need large metagenomic studies to taxonomise the gut microbiota and identify the species which confer protective vs damaging effects, and how they relate to each other.

Indeed, multiple environmental factors shape the gut microbiome in the first years of life, including geography and household exposures such as pets and siblings (131, 138). The ABIS study showed that exposure during pregnancy to cats and dogs conferred no increased risk of T1D, but hamsters did (139).

Obesity also negatively influences the gut microbiome. The obese adult individuals’ gut microbiome lacks diversity and the resultant dysbiosis, triggers immune dysregulation, inflammation and promotes diet-sustained obesity (140). The lack of diversity and composition in the gut of an obese individual is therefore similar to the T1D gut milieu. Consequently, there is research interest in interventions which negate these effects and help restore healthy gut microbiota. In mouse studies, faecal transplant from obese humans to germ free mice triggered greater weight gain, and the opposite also remains true. Allogenic healthy donor faecal transplant to individuals with metabolic syndrome improved insulin sensitivity and restored healthy gut flora (140). Trials of donor faecal transplant for T1D prevention have yet to be attempted, but de Groot et al. performed an RCT in new-onset T1D (<6 weeks) and found preservation of C-peptide following faecal transplantation (141).

In all cases, further studies need to address the range of factors which cultivate the gut microbiome, but dysbiosis appears to be an important hallmark for T1D pathogensis (142, 143).

### Breast Milk

TEDDY showed that the most significant determinant of gut taxonomy in the first year of life is breastfeeding (131). The World Health Organisation (WHO) recommend exclusive breastfeeding until ≥6 months of age, to support growth, development, immunity and the developing gut microbiome (144). However, practices differ across societies and cultures regarding duration of breast feeding, and the type and timing of solid foods (145). Unique benefits of breast feeding include transference of biologically active substances, such as antibodies, cytokines and hormones that modulate the developing immune system (130, 146). It is postulated that breast milk also confers protection from T1D through reduced frequency of infantile respiratory and gastrointestinal infections (147), delayed exposure to dietary antigens (gluten and bovine insulin) (12), and promotion of a healthy gut flora, seeding *Bifidobacterium* species (131). The DNBC and MOBA (29) population-based cohort studies, included 155,392 children and showed a two-fold increased risk [HR 2.29 (1.14-4.61)] of T1D in children not breastfed at 6-12 months compared to any breastfeeding for ≥12 months (30). There was no difference in T1D incidence between those fully or partially breastfed, and no association with age of introduction of solid foods (30). The MIDIA study explored breast feeding and age at introduction of solid foods with T1D risk in genetically susceptible children (148). Similarly, they found breastfeeding for ≥12 months predicted decreased risk of progression to T1D (HR 0.35 (0.13-0.94), with no effect on IA (148). Duration of full breastfeeding, age at introduction of solid foods and combination with breastfeeding, did not associate with risk of IA or T1D (148). Importantly, the prospective TEDDY study (149, 150) and the TRIGR RCT (151) showed no effect with duration of exclusive breastfeeding on seroconversion or progression to T1D. Despite the mixed results, ability to extrapolate further insights is limited, as the general health benefits of breastfeeding outweigh risk.

### Cow’s Milk and Formula Feeds

Cow’s milk, which contains bovine insulin, could potentially induce autoimmune responses through molecular mimicry to human insulin, leading to T1D seroconversion in children (152). A Finnish cohort study found that children exposed to cow’s milk formula before 3 months of age, had higher rates of IgG binding to bovine insulin antigen and these antibodies cross-reacted with human insulin (153); however none of these children went onto develop IA. The bovine insulin binding antibodies also inversely correlated with age at introduction of formula feed. Bovine insulin autoantibodies declined at 12 and 18 months, except in the anti-insulin antibody seropositive children, where levels significantly increased (153). The FINDIA study investigated bovine-insulin free formula feed with randomisation to three treatment arms (cow’s milk, whey-based hydrolysed formula and bovine-insulin free formula) and showed a reduced incidence of seroconversion in the bovine-insulin free formula feed group (32).

In light of concerns around introduction of cow’s milk (standard/conventional formulas), protein hydrolysed formula alternatives were trialled to determine risk reduction in T1D. In the Finnish TRIGR study, genetically susceptible children were randomised to either cow’s milk (CM) or casein-hydrosylate formula (CHF) feed, during the first 6-8 months of life where breast feeding was not possible, and found a reduced incidence of IA in the CHF group compared to CM group, with one [HR 0.51 (0.28-0.91)] or ≥two autoantibody positivity [HR 0.47 (0.19-1.07)] (154). The TRIGR study was a double-blind RCT including 2159 genetically at-risk children from 15 countries, followed-up for at least 10 years (33). TRIGR showed that weaning to hydrolysed formula compared with conventional formula (casein hydrosylate or adapted cow’s milk formula) did not decrease the cumulative incident risk of T1D after 11.5 years follow-up (33). Similarly, the TEDDY study generally showed no significant association between IA and hydrolysed or conventional formula feed (155). However, extensively hydrolysed formula feed was associated with an increased risk of IA when introduced in the first 7 days of life [HR 1.57 (1.04-2.38)] (155).

### Gluten

Coeliac disease is triggered by an autoimmune reaction to gluten, leading to villous atrophy in the small intestine and subsequent malabsorption (156). Coeliac disease affects 2.5% to 16.4% (5.7% overall) of individuals with T1D (157). Gluten is thought to trigger progression to beta-cell autoimmunity through molecular mimicry (158). The Finnish DIPP study, in 5545 genetical predisposed children, showed that higher intake of oats and gluten-containing foods increased risk of IA (159). The DAISY study showed cumulative gluten intake in the first 12 months did not associate with IA or T1D; however, introduction of gluten prior to 4 months of age significantly increased risk of T1D (160). On the contrary, the prospective TEDDY study showed that delaying introduction of gluten increased the risk of IA. Risk of developing islet antibodies was lower with introduction of gluten at <4 months of age compared to 4-9 months [HR 0.68 (0.47-0.99)], but higher compared to >9 months [HR 1.57 (1.07-2.31)] (161). However, TEDDY also showed that higher gluten intake in the first 5 years of life was associated with an increased risk for celiac disease (156).

Risks of other solid foods included in a weaning regimen have been explored but evidence is limited. The DIPP study linked early introduction of fruits, berries, and root vegetables, between 3-4 months of age, with increased risk of IA in genetically predisposed infants (162). Moreover, the TEDDY study showed protection against IA with introduction of egg, but the association was weak and did not remain when examined in a dose-response relationship (161). Virtanen et al. showed that early introduction of egg, at <8 months of age increased risk of IA in the first three years of life, but the relationship did not remain beyond 3 years follow-up (163).

Overall, we can deduce that introduction of solid foods presents a critical window to the gut microbiota, which may be protected by continuation of breastfeeding during this period (164, 165).

### Antibiotic Use

Antibiotics carry potential to chronically disrupt the gut microbiome, particularly in immunosuppressed individuals (130). The same concern applies to antibiotic treatment in early life, where new environmental exposures can shift microbial colonisation, conferring risk to T1D. Mikelson et al. (166) showed in a population case-control study that broad-spectrum antibiotic use in the first two years of life increased risk of T1D. A Finnish case-control study found T1D risk was associated with maternal pre-natal phenoxymethyl penicillin [OR 1.70 (1.08–2.68)] or quinolone use [OR 2.43 (1.16–5.10)] (167). Importantly though, antenatal antibiotic use did not affect risk. The UK Health Improvement Network (THIN) database revealed increased antibiotic exposure was associated with T1D risk, observed when taking 2-5 courses of cephalosporins [OR 1.41 (1.11–1.78)] or >5 courses of penicillins [OR 1.63 (1.26–2.11)] (168). However, the TEDDY study showed cumulative antibiotic use within the first four years of life did not associate with seroconversion [HR 0.98 (0.95-1.10)] or autoantibody progression [HR 0.99 (0.95-1.02)] (169). Similarly, Tapia et al. (170) showed no link between acetaminophen use in the first 6-9 months of life and risk of T1D in a Norwegian cohort.

### Probiotic Use

Agents which alter gut bacterial flora provide opportunities to restore a healthy microbiome for primary and secondary preventative purposes (171). However, evidence in support of their beneficial impact in reducing T1D risk is lacking. Probiotics consist of live micro-organisms and are engineered to restore healthy gut microbiota; protect gut membrane integrity; increase SCFA/butyrate production; reduce proinflammatory cytokines; and promote anti-inflammatory cytokines (171). In the TEDDY study, probiotic use in the first 27 days of life reduced risk of IA, compared to probiotic use after 27 days of life or no probiotic use, HR 0.66 (0.46-0.94), but this was only observed in genetically predisposed individuals (150). A double-blind placebo RCT compared maternal and infant probiotic supplementation in 1223 babies at risk of allergy and found no association with IA by 5 years, or overt T1D by 13 years, but this was a small sample size in a population not at risk of T1D (172). Prebiotics similarly aim to restore healthy gut flora and confer immunomodulatory benefits. Prebiotics comprise fructo-oligosaccharides, galacto-oligosaccharides, lactulose, or indigestible carbohydrates, are selectively up taken by gut microbiota and are associated with SCFA production, but have not been tested as a protective agent in T1D. Overall, evidence to support probiotics or prebiotics in the primary or secondary prevention of T1D is limited, but represent novel targets for therapeutic trials in genetically predisposed and seroconverted individuals (171).

### Vitamin D

Vitamin D is a candidate for protection against T1D due to its anti-inflammatory effects, role in regulation of the immune system and induction of T regulatory cells, which modulate autoimmune risk (8). Cathelicidin was recently proposed to link vitamin D with the gut microbiota and protective effects on beta-cell function (173). Further evidence stems from the higher incident cases of T1D observed at northern latitudes and in winter months compared to summer, where sunlight exposure inversely correlates with T1D cases on a monthly basis (174). However, studies exploring the relationship between vitamin D concentration and supplementation with T1D risk demonstrate mixed results (175–178).

A higher serum vitamin D reduces risk of IA, as demonstrated by a dose-response meta-analysis which found a U-shaped relationship with an OR 0.91 (0.90-0.93) for T1D per 10nmol/L increase in vitamin D (179). In contrast, the prospective DAISY study found no association between vitamin D concentration and seroconversion or T1D disease progression in IA positive individuals (175). This finding was corroborated by the prospective DIABIMMUNE study (180). Importantly however, the TEDDY study confirmed that higher plasma 25‐hydroxyvitamin D correlated with lower risk for IA in genetically predisposed children (181). More copies of the Vitamin D Receptor allele (VDR) due to a Single Nucleotide Polymorphism (SNP-86), conferred greater protection. Interestingly, dairy product vitamin D supplementation in Finland has since been associated with the stabilising incidence of T1D in this region (181).

Regarding supplementation, the Finnish birth cohort study found that in cases where the recommended dose was supplemented in the first year of life, >2000 units per day compared to <2000 units per day, relative risk (RR) for T1D was much reduced at 0.22 (0.05-0.89) (182). A Norwegian study showed that vitamin D and cod liver oil supplementation from 7-12 months of age reduced risk of T1D compared to supplementation from birth to 6 months of age (183). Further, the EURODIAB study showed that vitamin D supplementation in infancy was associated with reduced risk of T1D (184). A meta-analysis also showed a 29% (0.60-0.84) risk reduction for T1D with vitamin D supplementation (178). However, the DAISY prospective cohort did not identify an association between vitamin D and IA or risk of progression to T1D (175). In the ABIS study, infantile, intermediate vitamin D supplementation also did not associate with IA (176). Analysis of the TEDDY cohort for infantile vitamin D supplementation and T1D risk is awaited. However, the TEDDY study and a meta-analysis showed no association between maternal vitamin D supplementation and offspring’s T1D risk (177). The jury is out but further trials are warranted to further explore the value of vitamin D in the T1D risk story.

### Nicotinamide

Nicotinamide delays beta-cell failure enhances resistance to beta-cell toxins and increased regenerative capacities observed in NOD mice (185, 186). The ENDIT RCT investigated islet cell antibody (ICA) positive, first degree relatives of people with T1D but found no significant association with T1D (34). The DENIS study similarly showed no benefit with high dose nicotinamide at 3 years follow-up in genetically predisposed first-degree relatives (35).

### Omega-3 Poly-Unsaturated Fatty Acids

Omega-3 poly-unsaturated fatty acids (PUFA) reduce pro-inflammatory cytokines and may protect against T1D (187). Studies exploring benefit with omega-3 supplementation however have shown mixed results (188, 189). The TRIALNET Pathway to Prevention study compared omega-3 supplementation in the third trimester of pregnancy compared to infants aged 5 months and found no difference in pro-inflammatory cytokine profiles (188). In the DAISY study, Norris et al. identified a risk reduction in IA in infants supplemented with omega-3 PUFA from 12 months of age (189). This association was strongest in participants who were positive for more than 2 autoantibodies. The DAISY study further showed this increased risk was associated with reduced omega-3 PUFA in the red blood cell membranes. Reduced membrane concentration of docosapentaenoic acid predicted increased risk of IA and an individual’s genotype determined protective effects of α-linolenic acid supplementation (165, 190).

### The Gut – A Summary

The role of the gut microbiome and diet has been an area of active interest and research. There is strong evidence for an association between the microbiome (and factors that affect it) and T1D, and this is worth further exploration (129–132). However, the association of T1D with the many dietary agents that have been postulated remain to be confirmed and tested in an RCT setting.

## Discussion

Despite over 40 years of investigation, with multiple, international case-control, cohort, and prospective studies, we are still in search of those critical environmental triggers for T1D. The TEDDY study has provided the largest evaluation of environmental triggers in genetically predisposed children to date (26). Lessons learned are that T1D is a highly heterogenous condition, influenced by both genetic (13) and environmental factors (14), which interact through the threshold hypothesis (15, 26), to initiate and promote T1D over time.

We would suggest that a way forward for this field is first to explore and establish those environmental factors that probably associate with risk for IA and/or T1D. Once identified, they can then be tested, ideally through a RCT.

**Figure 1:**
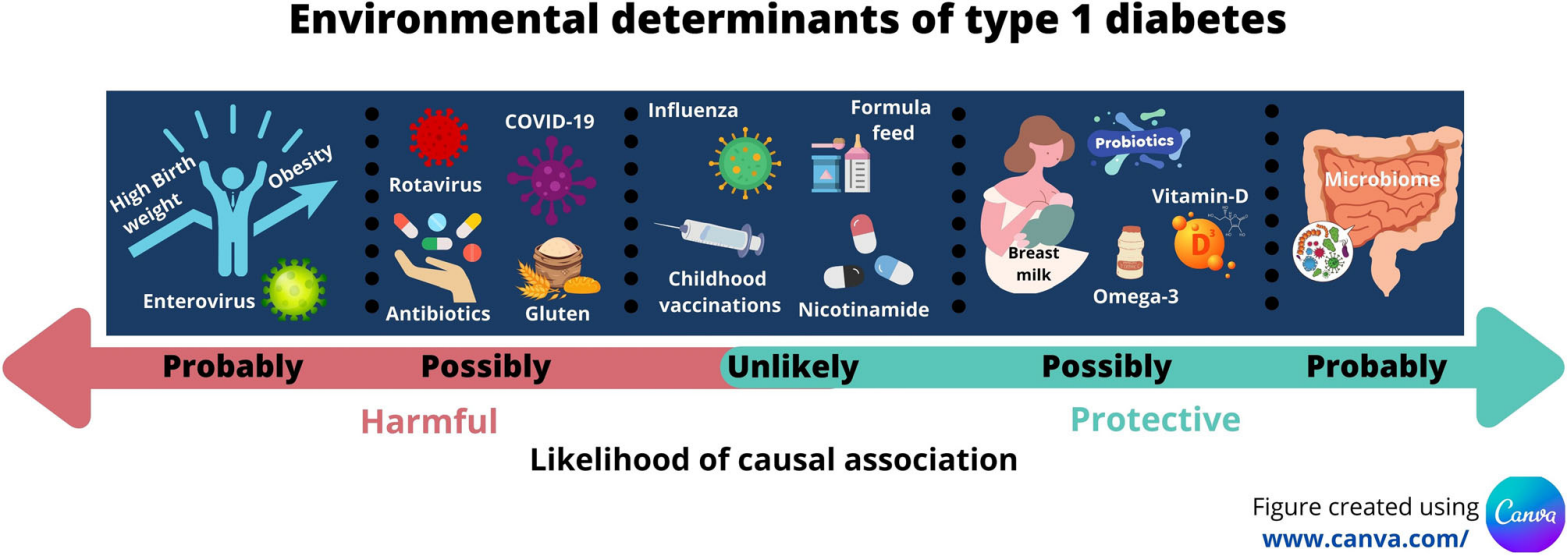
Infographic illustrating the key environmental determinants of type 1 diabetes and their likelihood of contributing to causality.

This proposal comes with challenges. The challenges of recruiting, defining and measuring exposure to the environmental agent, and allowing a sufficient period of follow up for IA and T1D to develop should not be under-estimated and has been outlined by others (191). Bearing these issues in mind, our review suggests probable associations with enterovirus infections; birth weight; early growth; childhood obesity; and with changes in the gut microbiome (**Table 1**). Several other possible associations exist but these need further evaluation. **Figure 1** summarises the likelihood of effect influenced by the environmental discussed in this review.

**Table 1:** List of the key environmental determinants outlined in this review and the evidence supporting a causal framework.

Class of agent	Agent	Current strength of association with IA or T1D	Proving contribution to causality	Supporting References
Infections and vaccinations	Enterovirus	Probable	Vaccination trials in planning	(48, 57)
	Rotavirus	Possible	Rotavirus vaccinations being incorporated into childhood vaccination programmes in some countries	(58, 65)
	Influenza	Unlikely	Studies show inconsistent results	(70–73)
	COVID-19	Possible	Vaccination programmes being set up	(37, 75–78, 87)
	Childhood vaccinations	Unlikely	Studies show inconsistent results	91, 92
Weight	Birthweight	Probable	RCT and intervention studies needed	(29, 111, 112)
	Infant growth	Probable	RCT and intervention studies needed	113, 114 (192)
	Childhood obesity	Probable	RCT and intervention studies needed	(115–119)
The Gut	Microbiome	Probable	RCT needed	(31, 129, 131–133)
	Breastfeeding	Possible	RCT evidence supports no role	(131, 151)
	Cow’s milk/formula feeds	Unlikely	RCT evidence supports no role	(32, 33, 153, 155)
	Gluten	Possible	Studies show inconsistent results	(156, 159–161)
	Antibiotic use	Possible	Studies show inconsistent results	(166, 168–170)
	Probiotic use	Possible	RCT evidence supports no role but small study	(150, 172)
	Vitamin D	Possible	Conflicting RCT results of vitamin D supplementation	(176–178, 182–184)
	Nicotinamide	Unlikely	RCT evidence supports no role	(34, 35)
	Omega-3 (PUFA)	Possible	Conflicting RCT results of PUFA supplementation	(188, 189)

The subsequent testing of ‘probable association’ also brings challenges. Some agents do not lend themselves easily to testing with a gold-standard RCT (birth weight and rate of childhood growth), and others cannot be tested because programmes to control the putative agent have been, or are being, implemented for other public health reasons (rotavirus, COVID-19) (88, 193). Yet other environmental agents such as childhood obesity may be considered unethical to test because there are good arguments for establishing a national programme to address this major global health burden (8). Proving causality for these agents will require means of assessment other than RCTs. However, well conducted RCTs, as was undertaken for the TRIGR study comparing hydrolyzed infant formula compared to cow’s milk-based formula (33), can be effective at addressing long-standing concerns about the T1D risk of particular environmental agents.

In conclusion, we present a summary of the environmental determinants according to the leading hypotheses; infection and vaccinations, the accelerator hypothesis, and the gut microbiome, and we outline the necessary routes to transition from association to causality.

## Author Contributions

LQ, FW, and PN made substantial contributions to the following: conception or design of the work; drafting the work or revising it critically for important intellectual content; providing approval for publication of the content; and agree to be accountable for all aspects of the work in ensuring that questions related to the accuracy or integrity of any part of the work are appropriately investigated and resolved. All authors contributed to the article and approved the submitted version.

## Conflict of Interest

The authors declare that the research was conducted in the absence of any commercial or financial relationships that could be construed as a potential conflict of interest.

## Publisher’s Note

All claims expressed in this article are solely those of the authors and do not necessarily represent those of their affiliated organizations, or those of the publisher, the editors and the reviewers. Any product that may be evaluated in this article, or claim that may be made by its manufacturer, is not guaranteed or endorsed by the publisher.
